# Depression during pregnancy: views on antidepressant use and information sources of general practitioners and pharmacists

**DOI:** 10.1186/1472-6963-9-119

**Published:** 2009-07-17

**Authors:** Tessa Ververs, Liset van Dijk, Somaye Yousofi, Fred Schobben, Gerard HA Visser

**Affiliations:** 1 Department of Clinical Pharmacy, University Medical Center Utrecht, Utrecht University, the Netherlands; 2 NIVEL – Netherlands Institute for Health Services Research, Utrecht, the Netherlands; 3 Department of Pharmacoepidemiology and Pharmacotherapy, Utrecht Institute for Pharmaceutical Sciences (UIPS), Utrecht University, Utrecht, the Netherlands; 4 Department of Perinatology and Gynaecology, University Medical Center Utrecht, PO Box 85500, 3508 GA Utrecht, the Netherlands

## Abstract

**Background:**

The use of antidepressants during pregnancy has increased in recent years. In the Netherlands, almost 2% of all pregnant women are exposed to antidepressants. Although guidelines have been developed on considerations that should be taken into account, prescribing antidepressants during pregnancy is still a subject of debate. Physicians and pharmacists may have opposing views on using medication during pregnancy and may give contradictory advice on whether or not to take medication for depression and anxiety disorders during pregnancy. In this study, we investigated information sources used by general practitioners (GPs) and pharmacists and their common practices.

**Methods:**

A questionnaire on the use of information sources and the general approach when managing depression during pregnancy was sent out to 1400 health care professionals to assess information sources on drug safety during pregnancy and also the factors that influence decision-making. The questionnaires consisted predominantly of closed multiple-choice questions.

**Results:**

A total of 130 GPs (19%) and 144 pharmacists (21%) responded. The most popular source of information on the safety of drug use during pregnancy is the Dutch National Health Insurance System Formulary, while a minority of respondents contacts the Dutch national Teratology Information Service (TIS). The majority of GPs contact the pharmacy with questions concerning drug use during pregnancy. There is no clear line with regard to treatment or consensus between GPs on the best therapeutic strategy, nor do practitioners agree upon the drug of first choice. GPs have different views on stopping or continuing antidepressants during pregnancy or applying alternative treatment options. The debate appears to be ongoing as to whether or not specialised care for mother and child is indicated in cases of gestational antidepressant use.

**Conclusion:**

Primary health care workers are not univocal concerning therapy for pregnant women with depression. Although more research is needed to account for all safety issues, local or national policies are indispensable in order to avoid undesirable practices, such as giving contradictory advice. GPs and pharmacists should address the subject during their regular pharmacotherapeutic consensus meetings, preferably in collaboration with the TIS or other professionals in the field.

## Background

Pregnancy is a vulnerable period, also when it comes to the adverse effects of drugs. Antidepressant use is increasing, including among women who plan to become pregnant or are pregnant [1,2]. It is not known how primary health care workers deal with the risks of treatment versus the risks of the disease and what sources of information they use on this topic. Treatment of depression and anxiety may consist of psychotherapy, medication, electroconvulsive therapy or a combination of several approaches. General practitioners (GPs) treat 86% of the patients with mental health problems themselves, and prescribe drugs in most cases [3].

Doctors are confronted with a novel situation when a patient becomes pregnant, a state in which all nonessential drugs should be avoided. However, pregnancy does not prevent depression, and its prevalence is estimated at between 14% and 20% [4].

Pharmacological treatment during pregnancy requires fine balancing of risks and expected benefits in each individual patient, taking the patients history, presentation and preferences into account. The safety of antidepressant use during pregnancy is still under debate since studies on risks of major malformations, persistent pulmonary hypertension and long term effects on neurodevelopment report conflicting results [5-8]. On the other hand, stress and depression are known to endanger both the mother and pregnancy outcome, including abnormal behavioural development of the infant at follow-up [9]. GPs are facing the dilemma of whether or not to prescribe antidepressants. They also have to consider other treatment options such as psychological therapies which have been proven to be effective in mildly to moderately depressed outpatients[10] Because of its relatively low prevalence, it is difficult for each individual GP to gain experience in this specific field. Therefore, it may be difficult for them to deal with this dilemma when they are not able to find useful information or apply the available data in practice [11,12]. In the Netherlands 2% of all pregnant women use antidepressants during pregnancy but another 2% stops using them [1]. Although fluoxetine is one of the antidepressants with the most published experience and sertraline is considered to have the lowest placental passage, pregnant women use all different kinds of antidepressants, including the ones that have come on to the market only recently [1].

We were interested in reasons for this variety in drug use. The source of information is an important factor that influences GPs' views on managing the mental health problems of pregnant women [13]. Inconsistencies between information sources may lead to contradictory views and, as such, may give rise to confusion. For instance, information from pharmaceutical companies is cautious and restricted to the contents of the summary of product characteristics (SPC), which mentions associations between antidepressant use during pregnancy and the risk of pulmonary hypertension, cardiovascular birth defects and neonatal withdrawal syndrome[14,15]. The Dutch national Teratology Information Service (TIS), which cites large database studies, reports that although the increased risk of major congenital malformations has to be considered, the absolute risk for individual patients remains small. Poor neonatal adaptation – which is usually mild and transient – may occur. On the other hand, it is not known whether fetal exposure to antidepressants has long-term effects on behaviour and neurodevelopment [9,16]. Finally, it is not known whether GPs follow practical guidelines or use local policies.

The role of the pharmacist – as a guardian and adviser on safe drug use during pregnancy – and the different sources of information that he/she explores compared to GPs have not been studied. We do not know whether GPs and pharmacists are aware of the specific risks of using antidepressants during pregnancy or the risks of refraining from medication, or whether they are acting accordingly.

It was the objective of this study to investigate where GPs and pharmacists in the Netherlands obtain information on the safety of gestational drug use and the pharmacotherapeutic approach when managing depression and anxiety during pregnancy.

## Methods

### Context

The University Medical Center (UMC) Utrecht is involved in a research project on the effects of antidepressants during pregnancy. As part of this, we studied treatment policies of the main prescribers of antidepressants, the GPs, and the reactions of pharmacists, who might intervene when a pregnant patient comes to the pharmacy with a prescription for an antidepressant. An inquiry was conducted in a Dutch population of 700 randomly selected GPs and 700 pharmacists. Permission of the ethics committee of the UMC Utrecht was not required to perform the investigation.

### Study design

We designed a questionnaire with items addressing policies in general practice in managing of depression and anxiety during pregnancy and the sources of information on this subject. We rated the participants' support for different pharmacotherapeutic approaches by referring to possible treatment options such as stepping down medication or switching to another antidepressant. We also inquired about their views on the first-choice antidepressant during pregnancy and on the question whether special care for the neonate is considered when antidepressants are used until delivery. Answering the 20-item questionnaire would take 15 minutes. The questionnaire and a pre-stamped return envelope were sent out by mail. Randomly selected addresses of eligible GPs were provided by NIVEL (Netherlands Institute for Health Services Research). Pharmacists were extracted randomly from members of the Royal Dutch Pharmaceutical Society (KNMP). The study was conducted in 2006.

We analysed differences in sex and practice characteristics between the two groups of respondents and compared them to the Dutch population of health care workers in the field.

### Statistical analysis

We used descriptive analyses (frequencies and cross tabulations). Differences between GPs and pharmacists were tested using chi-square tests. We did not correct for multiple testing. Data were analysed with SPSS, version 15.0.

## Results

A total of 132 GPs and 144 pharmacists returned the questionnaires, resulting in a response rate of approximately 20%. The general characteristics of all participants are presented in Table 1 and 2. Demographical differences between our sample and the entire Dutch population of GPs and pharmacists were negligible. Nevertheless their approach to treatment may differ.

**Table 1:** Characteristics of participating practitioners

		**General practitioners**		**Pharmacists**	
		**Participants** **(N = 130)**	**National** **(N = 8,209)**	**Participants** **(N = 144)**	**National** **(N = 2,789)**
**Sex**					
Man	%	63	66	53	54
Woman	%	37	34	47	46
					
**Practice**					
Solo	%	33	25	ne	ne
Duo	%	33	30	ne	ne
Group	%	34	45	ne	ne
					
**Patients**					
Mean	n	2758	4283	9148	8300
SD	n	1087	ne	3598	ne
					
**Working experience in same practice (years)**					
1–5	%	20	ne	26	ne
6–10	%	27	ne	30	ne
11–20	%	22	ne	28	ne
21–35	%	32	ne	15	ne

ne = not estimated

**Table 2:** Characteristics of the practice of participating practitioners

	**General practitioners** **(N = 130)**	**Pharmacists** **(N = 144)**	**Significant difference between GPs and pharmacists?**
**Number of pregnant patients annually**			*
0–20	57	21	
21–30	32	12	
31–100	32	40	
101–300	0	15	
Unknown	9	56	
			
**Written policy on treatment of depression and anxiety during pregnancy**			ns
Yes	1	3	
No	128	140	
Unknown	0	1	

*Presented is the number of pregnant patients visiting the practice annually and the availability of a policy on treatment of depression and anxiety during pregnancy in the practice of participation practitioners*.

*p < 0.01, ns = not significant

### Policy on "Depression and anxiety during pregnancy"

One of all GPs who responded had a local written policy on the treatment of depression and anxiety during pregnancy which was available in the practice (Table 2). For pharmacists, the results were comparable: although three pharmacists stated they had a local written policy, only one provided a copy. This particular policy recommended stepping down medication if possible. It mentioned fluoxetine and tricyclic antidepressants as the first choice, and recommended neonatal observation during the first two days after birth if drug use was continued until delivery.

### Information sources used in pharmacotherapy during pregnancy

Table 3 lists information sources used by GPs and pharmacists in decision-making and in advisory tasks when dealing with pregnant patients. A few participants (among both GPs and pharmacists) use standard works like *Drugs in Pregnancy and Lactation *by Briggs, Freeman and Yaffe [17]. Two thirds of GPs and a third of pharmacists never consulted the national TIS, which is a significant difference (chi^2 ^= 58.3; p < 0.001). Almost three quarters (72%) of GPs regularly consult pharmacists for information on drugs during pregnancy. Pharmacists, on the other hand, would not consult a specialist such as a psychiatrist, who is a specialist in the field. In the Netherlands, the reference used most frequently by both pharmacists and GPs is the "Pharmacotherapy Compass", the Dutch National Health Insurance System Formulary, issued annually (in Dutch: *Farmacotherapeutisch Kompas*, comparable to the *Physicians' Desk Reference *in the US). The introductory paragraph of each chapter discusses specific drug use during pregnancy. Updates on research as well as information provided by manufacturers are reviewed and followed by recommendations. The majority of the GPs (85%) rely on the information of the *Farmacotherapeutisch Kompas*.

**Table 3:** Information sources used by professionals when applying medication during pregnancy.

	**General practitioners** **(N = 128) %**				**Pharmacists** **(N = 142) %**				**Significant difference between GPs and pharmacists?**
	**Always**	**Most of the time**	**Some-times**	**Never**	**Always**	**Most of the time**	**Some-times**	**Never**	
Standard works on drug use in pregnancy and lactation	1	2	5	91	0	3	4	94	ns
National Teratology Information Service	5	11	17	66	16	12	39	33	**
Pharmacist for general practitioners/psychiatrist for pharmacists	5	23	45	28	0	3	27	70	ns
National Health Insurance System Formulary	48	37	13	3	23	23	38	16	**
NHG-standards*	7	23	38	31	5	8	37	51	**
Manufacturer	0	5	21	74	6	7	63	24	**
Internet e.g. Pubmed or Medline (research reports, issued guidelines)	2	9	34	55	2	8	39	51	Ns

* Guidelines issued by the Dutch College of General Practitioners (NHG)

** p < 0.01

ns = not significant

nr = not relevant, item concerns 2 questions, one for each group

Guidelines issued by the Dutch College of General Practitioners (NHG) – known as NHG standards – are used to a lesser extent. The NHG standard on the treatment of depression does not mention pregnancy, and the standard on pregnancy does not mention gestational depression and anxiety. A quarter of GPs (26%) contact the manufacturer of a specific drug for information. Pharmacists turn to the industry significantly more frequently (76%; chi^2 ^= 71.2, p < 0.001). Forty-five percent of GPs and 49% of the pharmacists use the internet to look for information on scientific evidence or reports from consensus groups. One out of five participants answered "yes" to the question of whether the subject of "treatment of depression and anxiety during pregnancy" has ever been covered during professional education courses.

### Views on the therapeutic management of depression and anxiety before and during pregnancy

The results show that GPs' opinions on how to manage depression and anxiety during pregnancy were not univocal (Table 4). One out of five GPs (21%) said they regularly refer patients to a psychiatrist, while the others sometimes or never do so. Some GPs (9%) state that they sometimes advise terminating the pregnancy when patients who use antidepressants become pregnant, which was also the case for 4% of the pharmacists. Within the professions, opinions on continuing medication, lowering the dose or stepping down varied widely. The majority of the respondents (92% of the GPs and 98% of the pharmacists) never or occasionally advised the patient to substitute the antidepressant drug used for another one. Substituting psychotherapy for medication – in order to prevent drug exposure to the child – was never advised by 55% of GPs and 24% of pharmacists; (chi^2 ^= 30.2; p < 0.01). Advice given to women who intend to become pregnant did not differ from advice given to pregnant women.

**Table 4:** Approach to female patients who use antidepressants.

	**General practitioners** **(N = 130) %**				**Pharmacists** **(N = 144) %**				**Significant difference**
	**Always**	**Most of the time**	**Some-times**	**Never**	**Always**	**Most of the time**	**Some-times**	**Never**	**between GPs and pharmacists?**
**Advise to a patient who uses antidepressants and states being pregnant**									
Refer to a psychiatrist	6	15	50	29	7	9	39	45	*
Advise to terminate pregnancy	0	0	9	91	0	1	3	96	ns
Step down and stop antidepressant	13	52	27	7	4	24	52	19	**
Continue antidepressants at mild symptoms	1	8	52	40	1	13	47	40	ns
Continue antidepressants at severe symptoms	14	36	36	13	19	35	26	20	ns
Change the used antidepressant for another	0	2	64	34	1	7	75	17	**
Lower the dose	0	16	40	44	1	10	57	32	**
Psychotherapy instead of antidepressants	3	9	33	55	3	20	53	24	**
**Advise to a patient who uses antidepressants and is planning to become pregnant**									
Refer to a psychiatrist	5	13	52	30	11	14	34	41	**
Advise to postpone pregnancy	2	13	63	23	2	3	28	67	**
Step down and stop antidepressant	10	52	32	6	6	30	49	15	**
Continue antidepressants at mild symptoms	1	9	45	45	2	7	54	37	ns
Continue antidepressants at severe symptoms	11	39	33	17	13	38	30	19	ns
Change the used antidepressant for another	0	4	62	34	2	8	71	20	*
Lower the dose	0	10	47	44	1	8	52	38	ns
Psychotherapy instead of antidepressants	6	6	44	44	5	25	55	15	**

Presented are the answers to the question what would be your advice to a patient who uses antidepressants and states that she is pregnant or is planning to become pregnant? * p < 0.05, ** p < 0.01, ns = not significant

### Arguments in favour of treating pregnant women with antidepressants

Most participants agreed with the statement that the need to treat maternal complaints with antidepressants outweighs possible drug-associated risks for the child (Table 5). Only 20% of GPs and 58% of pharmacists were aware of the negative effects of depression and anxiety on a child's development. On the other hand, only 4% of GPs and 35% of pharmacists believed antidepressants are not associated with an increase in the risk of birth defects. Sometimes an unstable social environment was given as a reason for not changing medication during pregnancy. Some participants said that they lacked the experience or background information for providing a proper answer on this topic.

**Table 5:** Reasons in favour of prescribing antidepressants during pregnancy and reasons against pharmacotherapy during pregnancy.

	**General practitioners** **(N = 130) n**	**Pharmacists** **(N = 144) n**	**Significant difference between GPs and pharmacists?**
**What are reasons for treating depression or anxiety during pregnancy with antidepressants?**			
Because the severeness of maternal complaints outweigh possible risks for the child.	124	116	ns
Because depression and anxiety may affect the child's neurological and behavioural development.	28	84	**
Because antidepressants do not increase the risk on birth defects	5	51	**
Other	24	2	**
Not responded	11	24	ns
**What may be the reasons for avoiding antidepressants during pregnancy?**			
Because depression and anxiety do not affect the course of pregnancy	13	20	ns
Because depression and anxiety do not influence the development of the child	12	7	ns
Because antidepressants may have negative effects on the unborn child	93	82	ns
Because antidepressants may cause withdrawal effects after birth	44	53	ns
Because antidepressants are not officially registered for use during pregnancy	39	26	ns
Because psychotherapy is as effective as are antidepressants	36	18	*
Other	9	0	ne
Not responded	17	34	ns

* p < 0.05, ** p < 0.01, ns = not significant, ne = not estimated

### Arguments against treating pregnant women with antidepressants

Only a few participants considered maternal depression and anxiety as such as causing no harm to the child, thus antidepressants could be avoided in pregnancy (Table 5). Most GPs and pharmacists mention possible negative effects on the child as a reason to avoid antidepressants. For one third of GPs and pharmacists a possible neonatal withdrawal syndrome was the most important reason to avoid antidepressants during pregnancy. The fact that none of the antidepressants are registered for use during pregnancy was a reason to avoid antidepressants for 30% of GPs and for 18% of the pharmacists. Only 28% of GPs and 13% of pharmacists believe that antidepressants should be avoided because psychotherapy is just as effective as antidepressants.

### First-choice antidepressant during pregnancy

**Figure 1:**
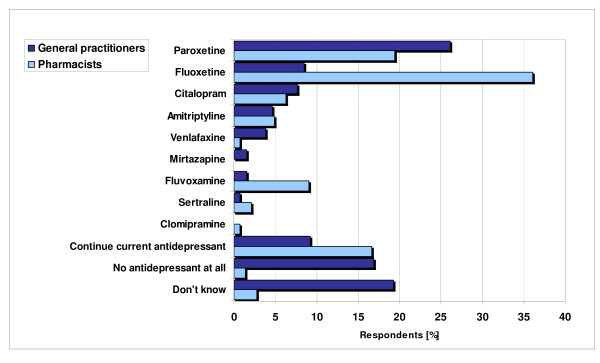
**Views on first choice pharmacotherapy during pregnancy**.

Nine different compounds were mentioned as the antidepressant of first choice during pregnancy (Figure 1). Paroxetine and fluoxetine were mentioned most frequently by GPs; fluoxetine was the favourite among pharmacists. Almost 20% of the GPs answered "no antidepressant at all" and another 20% stated they had no idea, or that they had no idea because of the absence of guidelines. Their reason for choosing a specific antidepressant was usually based on the first choice of their local pharmacotherapeutic consensus groups. Coming in second, they chose a drug because it had the most evidence of being safe to use during pregnancy. However, a minority of GPs (9%) and 5% of the pharmacists still consider St. John's wort to be a good alternative; one out of four participants saw no harm in using valerian during pregnancy (Table 6). There were no significant differences between the two groups of professionals.

**Table 6:** Views on the question *"Do you consider herbal drugs a safe alternative for use during pregnancy?"*

	**General practitioners** **(N = 128) %**	**Pharmacists** **(N = 143) %**	**Significant difference between GPs and Pharmacists?**
**St. Johns worth**			
Yes	9	5	ns
No	91	95	
**Valerian**			
Yes	21	24	Ns
No	79	76	

ns = not significant

### Is special perinatal care necessary?

A total of 13% of the responding GPs and 7% of the pharmacists did not believe that antidepressant use during pregnancy created a situation in which mother and child needed to receive special or additional perinatal attention (chi^2 ^= 13.1, p < 0.01). Another 36% of the GPs and 38% of the pharmacists did not consider antidepressant use to be an indication for delivery under the care of a gynaecologist (Table 7).

**Table 7:** Views on the question *"Do pregnant women who use antidepressants need special perinatal attention?"*

	**General practitioners** **(N = 130) %**	**Pharmacists (N=144) %**	**Significant difference between GPs and pharmacists?**
**Are regularly monitoring of the child with ultrasound, blood levels of antidepressants or follow-up on mental status of the mother required?**			
Yes	79	92	*
No	13	7	
Unknown	8	1	
**Is antidepressant use an indication for delivery in a hospital under the supervision of a specialist?**			
Yes	55	59	Ns
No	36	38	
Unknown	9	3	

* p < 0.05, ns = not significant

## Discussion

We investigated the opinions of general phycicians and their attitudes towards drug treatment of depression during pregnancy. In medical practice the risks and benefits of each individual patient will be weighed carefully, taking all other aspects such as psycho-social environment in to account. That made it difficult to give general answers to all the questions. Nevertheless, from the answers given we still can draw some conclusions. [13]. Although indications for prescribing antidepressants in general practice have been studied extensively and national as well as local guidelines and policies are issued, no specific attention has been given to pregnant women [18]. Considering the number of pregnant women who visit GPs and pharmacists annually and the prevalence of antidepressant use during pregnancy (2% to 8%), doctors and pharmacists will be confronted with this subject a number of times each year. They do not have a broad experience compared to experts in the field, but are the ones who decide on antidepressant use. The long waiting lists for psychotherapy in the Netherlands may influence a preference for pharmacotherapy.

Patients complain about receiving contradictory counselling, which appears to result from health care professionals using different sources of information [13]. Some follow the advice of pharmaceutical companies not to prescribe antidepressants to pregnant women or only when the benefits outweigh the risks. Package inserts of some antidepressants contain information about associations between antidepressant use during pregnancy and the risk of pulmonary hypertension, cardiovascular teratology and neonatal withdrawal syndrome. At the same time the national TIS may point out that antidepressant use during pregnancy has not been consistently shown to significantly increase the risk of major congenital malformations, they may warn of relapse of the illness and the negative effects of maternal stress and depression on the developing child, and may conclude that treatment should not be discontinued without thorough consideration. A total of 66% of GPs do not use this free national service. It is also surprising that while GPs value pharmacists as professionals who can be consulted on drug use during pregnancy, pharmacists in turn seldom consult psychiatrists, who may be more experienced on the subject when it comes to medication for mental illness during pregnancy.

From our results it can be concluded that there are large differences in views on managing depression before and during pregnancy. Extreme answers such as advising termination of the pregnancy or never switching to an antidepressant which is more likely to be safe in pregnancy show that professionals lack knowledge on the subject. It also may explain why such a varied pattern of antidepressant use exists. Although St. John's wort is a powerful herb with strong serotonergic effects, GPs and pharmacists are not unanimously opposed to its gestational use. It is not recommended during pregnancy since serotonin is not only a neurotransmitter but also a growth factor for the brain and other tissues [16]. Moreover the quality of herbal drugs is not always standardised. Valerian should also be used with caution. Hepatic failure during pregnancy as well as cytotoxic effects have been described [19].

When asked to consider the risks and benefits of antidepressant use during pregnancy, most but not all responders point out that although antidepressants may have negative effects on the child, the consequences of the mother's illness outweighs the possible risks. We failed to inquire after the option of continuing antidepressants when the mother had no symptoms. Most respondents underestimate the powerful and lasting effects of psychotherapy, and therefore deny patients a chance to cut down on medication. Not all GPs and pharmacists share the opinion that antidepressants could cause very serious withdrawal effects, which is probably the reason why more than a third of respondents think a delivery under the care of a midwife or GP is appropriate, while others acknowledge the need for carefully monitoring the baby after birth. The patients who participated in our research projects complain about receiving contradictory advice on this subject, considering the fact that almost half of Dutch mothers prefer to deliver at home [20].

### Limitations

Response rates in studies inquiring into policies are often disappointingly low, because in general practitioners give priority to direct care rather than to participating in research. The 20% response rate in this survey is in line with this. The professionals who returned the questionnaire might be the ones who are most involved with the subject. But then again, our conclusion that information and guidelines should be made available would hold true to an even greater extent. Part of our questions forced the respondents to choose among universal statements without weighing individual nuances. Nevertheless the answers revealed broadly deviating opinions.

## Conclusion

The data presented reflect the views of individual GPs and pharmacists on how to provide pregnant women with the best treatment, since few had policies they could refer to. Opinion pieces and literature reviews almost always end by stating that when treating pregnant women with antidepressants, the expected positive effects must be weighed against the risks. Doctors are then faced with a problem, though, because they do not have easy access to information on the safety and efficacy of antidepressants during pregnancy, which they could use to make an evidence-based decision. The more so, since most research data that can be found on the internet do not cover the entire range of possible short-term and long-term effects nor do they account for additional risk factors such as smoking or alcohol use. Therefore the Teratology Information Center – which evaluates the latest reports and collaborates on exposure studies – should be consulted more often, also since they have counselling services available for individual cases. Also, pharmacists – who seem to be credited to a great extent as being the ones to turn to when it comes to use of medication during pregnancy – could play an important role by initiating local policy meetings, providing easily accessible and interpretative information and reviewing guidelines. Development and implementation of clear policies will mean that pregnant women will no longer be sent from pillar to post.

## Competing interests

The authors declare that they have no competing interests.

## Authors' contributions

TV and GV: conception and design of the study. TV SY and LD analysed the data. GV and FS: supervised all aspects of the study, provided feedback and suggestions throughout. All authors helped to interpret findings and review drafts of the manuscript

## Pre-publication history

The pre-publication history for this paper can be accessed here:


